# Age- and sex-related changes in fasting plasma glucose and lipoprotein in cynomolgus monkeys

**DOI:** 10.1186/s12944-016-0280-x

**Published:** 2016-06-24

**Authors:** Feng Yue, Guodong Zhang, Rongping Tang, Zhouquan Zhang, Liqiong Teng, Zhiming Zhang

**Affiliations:** Department of Neurobiology, Beijing Institute of Geriatrics, Xuanwu Hospital of Capital Medical University, Beijing, 100053 China; Wincon TheraCells Biotechnologies Co., LTD, 3 Gaoxin 3 Rd, Nanning, 530003 Guangxi China; Department of Bio-engineering, Guangxi Medical University, 22 Shuangyong Rd, Nanning, 530021 Guangxi China; Guangxi Dongya Center for Nonhuman Primate Research and Technical Development, 3 Gaoxin 3 Rd, Nanning, 530003 Guangxi China; Department of Anatomy and Neurobiology, University of Kentucky College of Medicine, Lexington, KY 40536 USA

**Keywords:** Aging, Diabetes, Lipoprotein, Glucose, Sex, Cynomolgus monkey

## Abstract

**Background:**

The age-related dysfunction of glucose and lipid metabolism has a long-standing relationship with cardiovascular and neurodegenerative disease. However, the effects of metabolic dysfunction on men and women are different. Reasons for these sex differences remains unclear. Cynomolgus monkeys have been used, in the past, for the study of human metabolic diseases due to their biologically proximity to humans. Nevertheless, few studies to date have focused on both age- and sex-related differences in glucose and lipid metabolism. The present study was designed to specifically address these questions by using a large cohort of cynomolgus monkeys (*N* = 1,399) including 433 males and 966 females with ages ranging 4 to 24 years old.

**Methods:**

Fasting plasma glucose (FPG) and lipid parameters including total cholesterol (T-Cho), triglyceride (TG), high-density lipoprotein cholesterol (HDL-C) and low-density lipoprotein cholesterol (LDL-C) were measured. All these parameters were compared between ages and sexes.

**Results:**

Among the entire cohort, age was strongly correlated with levels of FPG, TG and HDL. Consequently, sex-related analysis revealed that females had significantly higher average levels of FPG, T-Cho, TG, HDL-C and LDL-C than their male counterparts. In addition, more female (28.5 %) than male (16 %) monkeys qualified for impaired fasting plasma glucose (IFPG). In those IFPG animals, sex-related differences were also detected i.e. females had significantly increased levels of T-Cho, TG and LDL-C.

**Conclusions:**

The result, for the first time, demonstrated the similarities and differences in detail between male and female cynomolgus monkeys in relationship to age-related glucose and lipoprotein metabolisms, and differences under various physiological conditions. The detailed glucose and lipoprotein profiling should provide additional and important insights for prediabetic conditions. Cynomolgus monkeys appear to be an excellent model for translational research of diabetes and for novel therapeutic strategies testing to overt diabetes.

## Background

National Diabetes Statistic Report 2014 reports that 37 % of U.S. adults aged 20 years or older had prediabetes (51 % of those aged 65 years or older). Applying this percentage of the entire U.S. population in 2012 yields an estimated 86 million Americans aged 20 years or older with prediabetes (http://119.90.25.23/www.cdc.gov/diabetes/pubs/statsreport14/nationaldiabetes-report-web.pdf). The report also revealed that the number of people with diabetes was 28.9 million in 2012, including the 4.3 million aged from 20–44, the 13.4 million from 45–64 and the 11.2 million from 65 years or older. If the total number of people with diabetes was divided by gender, men were 15.5 million and women were 13.4 million. In fact, among the 28.9 million people, or 9.3 %, that make up the U.S. population that are afflicted with diabetes, 8.1 million people are undiagnosed. Therefore, it is unclear how to prevent, best manage and treat metabolic diseases, particularly how to manage diabetes with multiple comorbidities and other geriatric complications [1, 2]. Nonhuman Primates (NHPs) are increasingly used in scientific and translational research mainly because of their similarity to humans in physiology, neuroanatomy, reproduction, development, cognition, and social complexity; thus confirming that one can address questions using NHP models that cannot be addressed using other species. For examples, cynomolgus monkeys are well-characterized animal models for investigating sex differences in susceptibility to diet-induced atherogenesis [3]; and age-associated changes of amyloid-β in cerebral spinal fluid [4].

Similar to humans, cynomolgus monkeys have an increased propensity to developing diabetes, particularly type 2 diabetes mellitus (T2DM) relative to increasing age [5–7]. This natural occurrence of T2DM has been reported in this kind of old-world primate by different laboratories in the past [8–11]. However, prediabetic conditions, which are associated with increased health risks, have been under investigation especially with a large cohort. Age- and sex-associated differences in fasting plasma and lipoproteins have not been described in detail in particular in nonhuman primates. The third guideline from the Adult Treatment Panel of the US National Cholesterol Education Program (ATP-III) recommends a full fasting lipoprotein profile, including triglyceride (TG), total cholesterol (T-Cho), high-density lipoprotein cholesterol (HDL-C), and low-density lipoprotein cholesterol (LDL-C) [12].

The present study was designed to characterize age- and sex-related changes in FPG and fasting lipoprotein in large groups of male and female cynomolgus monkeys with age raging from 2–24 years old. In humans, it is a challenge to study age- and sex-related differences due to the presence of multiple confounders that may directly or indirectly impact upon the outcome such as life style, dietary composition, physical activity etc. In nonhuman primates, they live in a laboratory-controlled environment provide a milieu where most of these factors are absent or tightly regulated particularly the daily diet and food supplement. Since cynomolgus monkeys are likely to provide an excellent model for studying prediabetic and diabetic conditions; results from the present study could supply important insights for novel drug testing designed to specifically address prediabetic condition, which is a factor for health.

## Methods

### Animals

Blood samples were randonmly collected from 1,399 cynomolgus monkeys (*Macaca fascicularis*) from a commercial vendor (Grandforest Primate Breeding Co. Ltd., Guangxi, China) during the past 3–4 years. They included 433 males and 966 females with ages ranging from 4 to 24 years old. Body weights at the time of sampling were ranging from 2.2 to 13.86 (mean = 5.34 ± 1.96) kilograms. Prior to each sampling sessions, informed consent from the vendor was obtained, and all procedures were conducted in compliance with animal care and use protocols. The animals were housed outdoors in big cages (9–15 animal/cage) and were fed a custom-in-house made pellet which is grain-based diet that contains ~ 20 % protein, ~5 % fat, and 8.5 % fibers and the remaining as carbohydrate and minerals at 150 g/monkey/day along with water available *ad libitum,*. In addition, all monkeys are supplemented with fresh fruits and vegetables which are rich source of vitamin C. The study protocol was reviewed and finally approved by the Institutional Animal Care and Use Committee (IACUC) of Wincon TheraCells Biotechnologies Co., Ltd. (Wincon) in Nanning Guangxi China, which is fully accredited by the Association for Assessment and Accreditation of Laboratory Animal Care (AAALAC).

### Animal husbandry

Cynomolgus monkeys used for this project were regularly group-housed outdoors in 15 m × 24 m open pens with 8 separate covered feeding stations to facilitate food and supplement accessibility. The animals were transferred from outdoors facility to single primate cage with the dimension of 28 in. depth, 39 in. width and 33 in. height for each cage, and were acclimated for at least 48 h after transfer. Leftover monkey chow was cleared from each cage before 18:00 PM, and blood sampling started after 8:00 AM the next morning.

### Blood sample and measurement

After the 14-h fasting, animals were anesthetized with 10 mg/kg ketamine and weighted after complete sedation. Four-microliters of blood was obtained from the femoral vein either in the left or right leg with a 20-gauge syringe, and transferred into an EDTA anticoagulant tube for each animal by well-trained lab technicians. The blood was immediately centrifuged at 3,000 rpm for 15 min, and the plasma was then stored at -20C°, which was then analyzed within 48 h. After sampling, most animals were put back to their group colony however a few were taken to the primate facility of Wincon to avoid desertion and inapt to the original groups.

Fasting plasma glucose (FPG), total cholesterol (T-Cho), triglyceride (TG), HDL-C and LDL-C were measured using the Hitachi 7600 Biochemical Analyzer (Hitachi Ltd., Tokyo, Japan) in a clinical laboratory, which is an affiliated hospital of Guangxi Medical University. All assay kits were manufactured by Shanghai Zhicheng Biological Technology Co., Ltd., FPG concentrations were measured using GOD-PAP method; Fasting T-Cho concentrations were measured using CHOD-PAP method; Fasting TG concentrations were measured using GPO-PAP method and Fasting HDL-C and LDL-C concentrations were measured using direct clearance method.

### Statistical analysis

All data was analyzed using Prism 6 GraphPad Software (San Diego, CA) and presented as mean ± SEM. The distribution of age was assessed by a one-way analysis of variance (ANOVA), followed by a multiple comparisons test. For normal Gaussian distribution, a one-way ANOVA was followed by Holm-Sidak’s multiple comparisons test; if normal distribution did not occur it was followed by Kruskal-Wallis test which is then followed by Dunn’s multiple comparisons test. In multivariate analyses, linear regression and/or Pearson Correlation for Gaussian distribution) or Spearman (for nonparametric) correlation coefficients (*r*) were used to describe the association between independent variables of interest. Differences between male and female were analyzed by unpaired student t-test or Mann-Whitney test (nonparametric test). A *P* value <0.05 was considered statistically significant for all analyses.

## Results

### Age-related differences

**Fig. 1:**
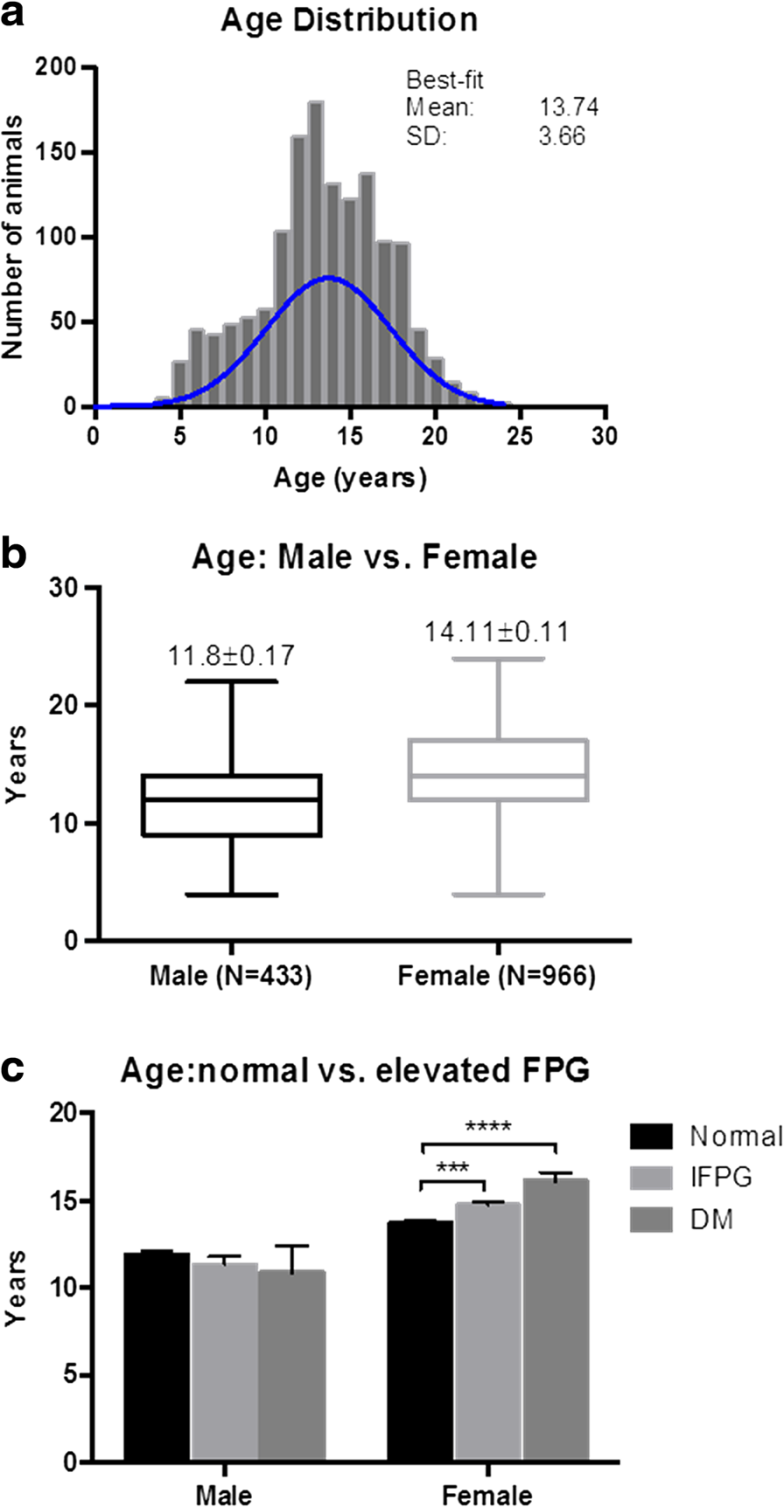
Age distribution and associated differences

**Fig. 2:**
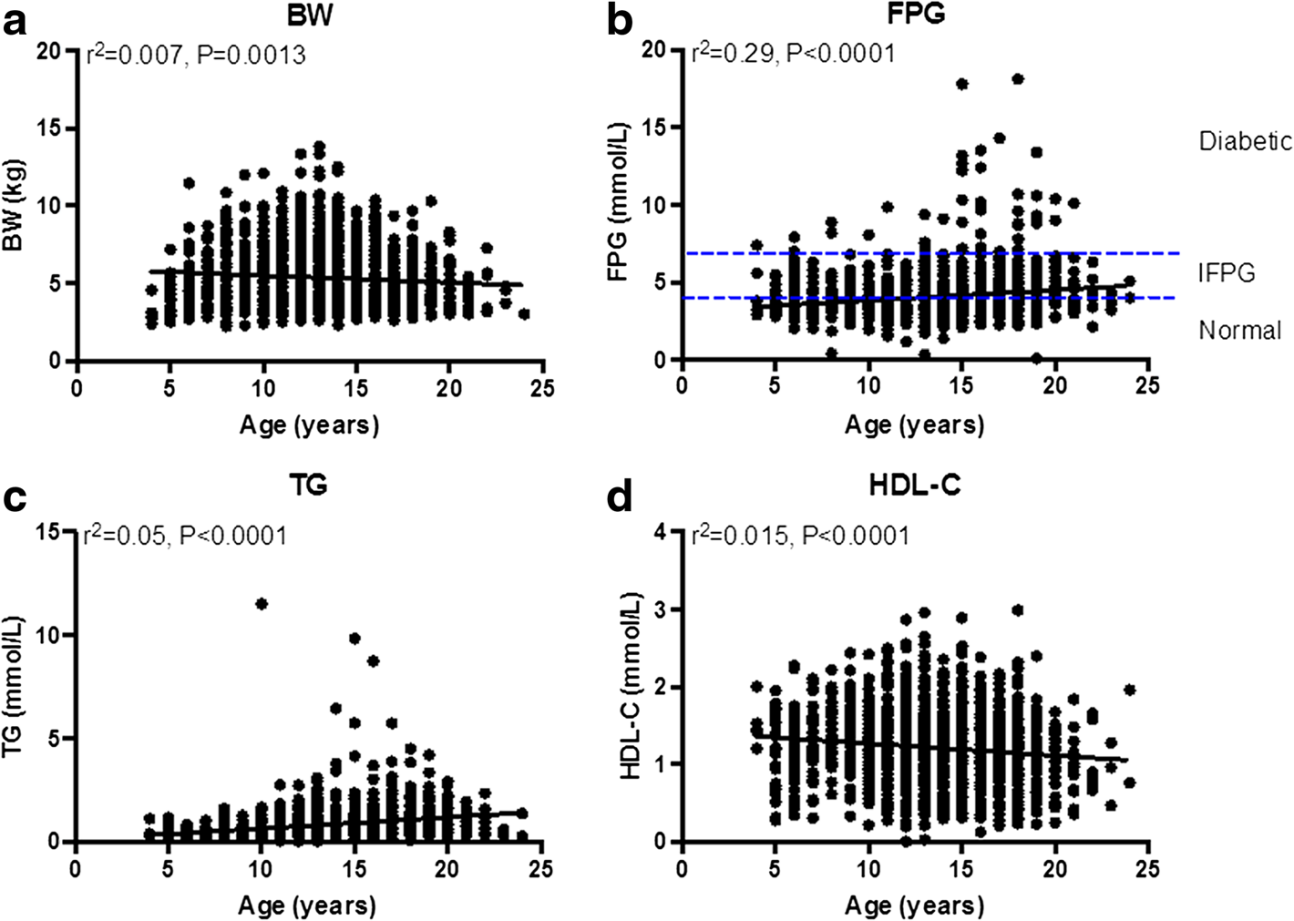
Age-associated changes in FPG and lipids

The average age of the cohort of animals was 13.74 ± 0.1 (Mean ± SEM) years old (Fig. 1a). Females (14.1 ± 0.11) were ~2.3 years older than males (11.8 ± 0.17) (Fig. 1b). When dividing animals by sex and their levels of FPG showed no difference between normal, IFPG and diabetic male animals. By contrast, significant differences between the normal and IFPG, and normal and diabetic animals were found in female monkeys (Fig. 1c). The age-related changes with metabolic parameter were summarized in Table 1. Examples of those correlations analyzed with linear regression were shown in Fig. 2.

**Table 1:** Pairwise correlations (*n* = 1,399)

	Age		BW		T-Cho		TG		HDL-C		LDL-C		FPG	
	*r*	*P* value	*r*	*P* value	*r*	*P* value	*r*	*P* value	*r*	*P* value	*r*	*P* value	*r*	*P* value
Age			−0.08	0.0013	−0.005	0.89	0.26	<0.0001	−0.12	<0.0001	0.012	0.67	0.17	<0.0001
BW	−0.08	0.0013			−0.27	<0.0001	0.045	0.097	−0.14	<0.0001	−0.26	<0.0001	−0.11	<0.0001
T-Cho	−0.005	0.89	−0.27	<0.0001			0.13	<0.0001	0.715	<0.0001	0.75	<0.0001	0.115	<0.0001
TG	0.26	<0.0001	0.045	0.097	0.13	<0.0001			−0.175	<0.0001	−0.019	0.45	0.28	<0.0001
HDL-C	−0.12	<0.0001	−0.14	<0.0001	0.715	<0.0001	−0.175	<0.0001			0.43	<0.0001	0.015	0.55
LDL-C	0.01	0.67	−0.26	<0.0001	0.75	<0.0001	−0.019	0.45	0.43	<0.0001			0.04	0.101
FPG	0.17	<0.0001	−0.11	<0.0001	0.115	<0.0001	0.28	<0.0001	0.015	0.55	0.04	0.101		

Pearson correlation was used with two-tailed *P* value. *BW* body weight, *T-Cho* total cholesterol, *TG* triglyceride, *HDL-C* total high-density lipoprotein, *LDL-C* total low-density lipoprotein, *FPG*, fasting plasma glucose

### Glucose and lipid profiles

Ages were highly correlated with FPG (Fig. 2b, Table 1). According to the American Diabetes Association plasma glucose level criteria for normal humans (Diabetes care., 2003), was identified as < 4.4 mmol/L, impaired fasting plasma glucose (IFPG) as 4.4-6.99 mmol/L and diabetic as > 6.99 mmol/L. Based on this diagnostic criteria, 1,003 out of 1,399 (~71.7 %) were qualified as normal, 344 out 1,399 (~24.6 %) were IFGP and 47 out of 1,399 (~7 %) were diabetic. Remarkably, some IFPG monkeys were as young as 4 years old, although most IFPG animals were found between 15–20 years old (Fig. 2b). Other more interesting correlations included ages that were positively correlated with levels of TG while negatively with levels of HDL-C (Fig. 2c, d). No changes were found in levels of T-Cho and LDL-C over aging (Table 1).

### Sex-related differences

**Fig. 3:**
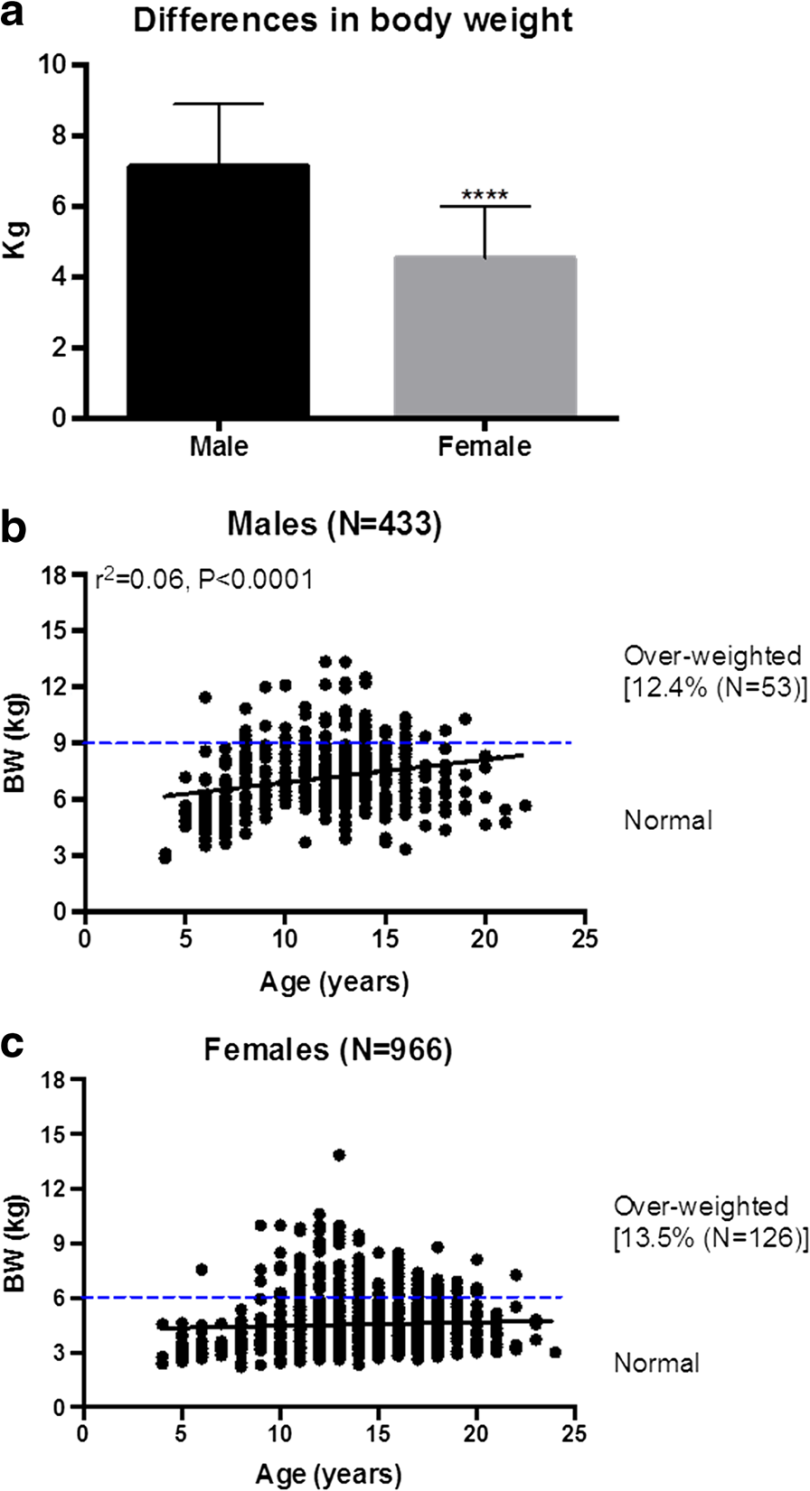
Sex-related differences in bodyweight

Male’s body weights were significantly (*P* < 0.001) heavier than the female’s on the average (Fig. 3a). In males, body weights increased with aging (Fig. 3b) while virtually no changes were seen in females (Fig. 3c). However, the overall correlation between age and body weight in this cohort of monkeys showed a slightly downward trend (*r* = −0.08, *P* = 0.0013) (Table 1). Established data suggest that the normal weight of cynomolgus monkeys are between 3.5 and 9Kg for males and 3 and 6Kg for females, which was displayed in the current study [13]. Based on the standard, the percentage of over-weighted animals was 12.4 % in males and 13.5 % in females respectively (Fig. 3b, c).

### Glucose and lipid profiles

**Fig. 4:**
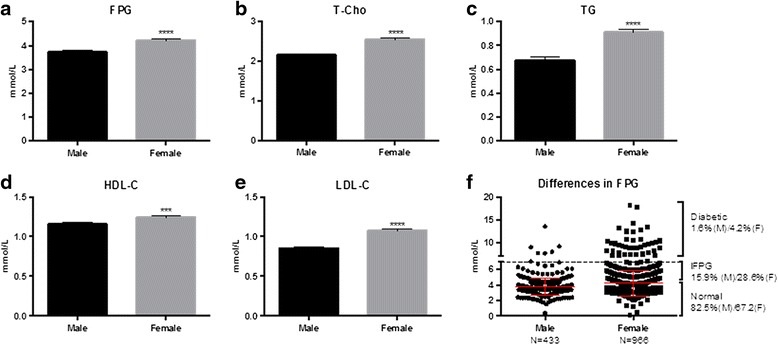
Sex-associated differences in FPG and lipids

**Fig. 5:**
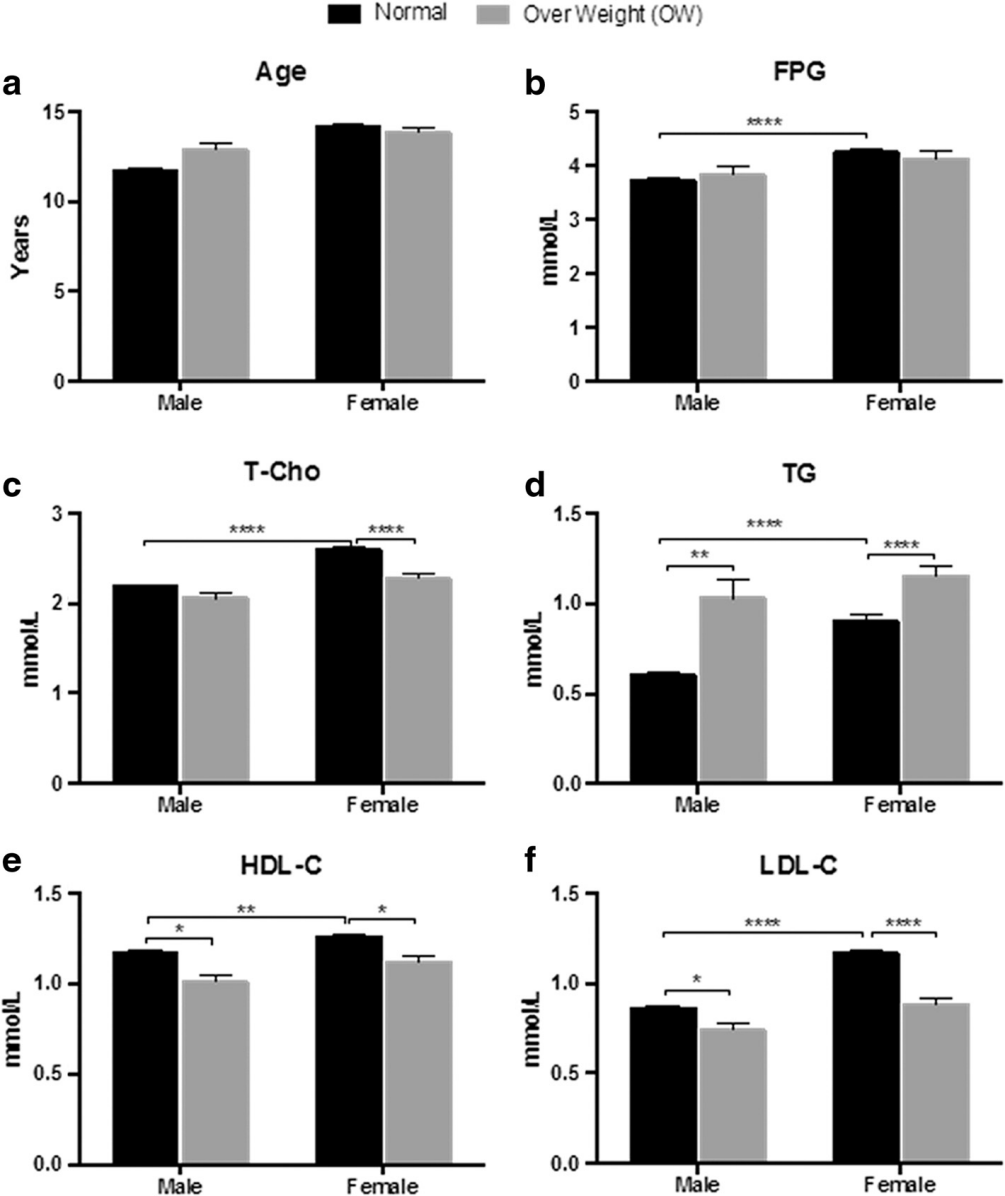
Differences between normal and overweight animals

Among this cohort of animals, females depicted higher levels of all measured parameters including FPG, T-Cho, TG, HDL-C and LDL-C than male monkeys (Fig. 4a-e). If a focus was aimed to levels of FPG, in particular, it would not be difficult to identify more female monkeys qualified with IFPG and diabetes in percentage than their male counterparts (Fig. 4f). When comparing between normal and overweight animals, no statistical significances were found in age and FPG (Fig. 5a, b).

If the focus was in between normal and IFPG animals, as summarized in Table 2, several major findings between sexes under the two physiological conditions were: 1) no statistical significance was found in levels of FPG between normal male and female or between IFPG male and female monkeys; 2) higher levels of T-Cho, TG and LDL-C were found in female monkeys under both normal and IFPG conditions than those of the males; 3) higher level of HDL-C were found in the normal females than that of the males; while no differences were found between IFPG male and female monkeys.

**Table 2:** Differences between normal and IFPG animals

	Normal			IFPG			Difference	
	Male	Female	*P* value	Male	Female	*P* value	*P* value^1^	*P* value^2^
	(*N* = 357)	(*N* = 648)		(*N* = 69)	(*N* = 275)			
Age (years)	11.91 ± 0.18	13.72 ± 0.14	****	11.32 ± 0.48	14.76 ± 0.21	****	*ns*	**
BW (Kg)	7.14 ± 0.08	4.58 ± 0.08	****	7.12 ± 0.24	4.53 ± 0.08	****	*ns*	*ns*
FPG (mmol/L)	3.38 ± 0.03	3.45 ± 0.02	*ns*	5.02 ± 0.07	5.16 ± 0.04	*ns*	****	****
T-Cho (mmol/L)	2.15 ± 0.02	2.52 ± 0.03	****	2.27 ± 0.06	2.63 ± 0.04	***	ns	*ns*
TG (mmol/L)	0.65 ± 0.02	0.86 ± 0.05	****	0.77 ± 0.07	1.01 ± 0.04	**	ns	****
HDL-C (mmol/L)	1.14 ± 0.02	1.22 ± 0.02	*	1.24 ± 0.05	1.31 ± 0.03	*ns*	ns	*ns*
LDL-C (mmol/L)	0.85 ± 0.02	1.07 ± 0.02	****	0.85 ± 0.03	1.12 ± 0.03	***	ns	*ns*

Results are presented as mean ± SEM. Kruskal-Wallis test followed by Dunn’s multiple comparison test. ns, not significant. *, *P* < 0.05; **, *P* < 0.01; ***, *P* < 0.001; ****, *P* < 0.0001. *BW* body weight, *FPG* fasting plasma glucose, *T-Cho* total cholesterol, *TG* total triglyceride, *HDL-C* total high-density lipoprotein, *LDL-C* total low-density lipoprotein

1. Comparing between normal and IFPG in males

2. Comparing between normal and IFPG in females

When comparing between normal and over-weight monkeys, levels of TG were significantly higher in over-weight for both male (*P* < 0.01) and female (*P* < 0.001) monkeys (Fig. 5d. Contrarily, significantly lower levels of HDL-C (*P* < 0.05 for both sexes) and LDL-C (*P* < 0.05 for male and *P* < 0.0001 for female) were found in over-weight animals (Fig. 5e, f); and lower levels of T-Cho (*P* < 0.0001) were only seen in female over-weight animals (Fig. 5c).

**Fig. 6:**
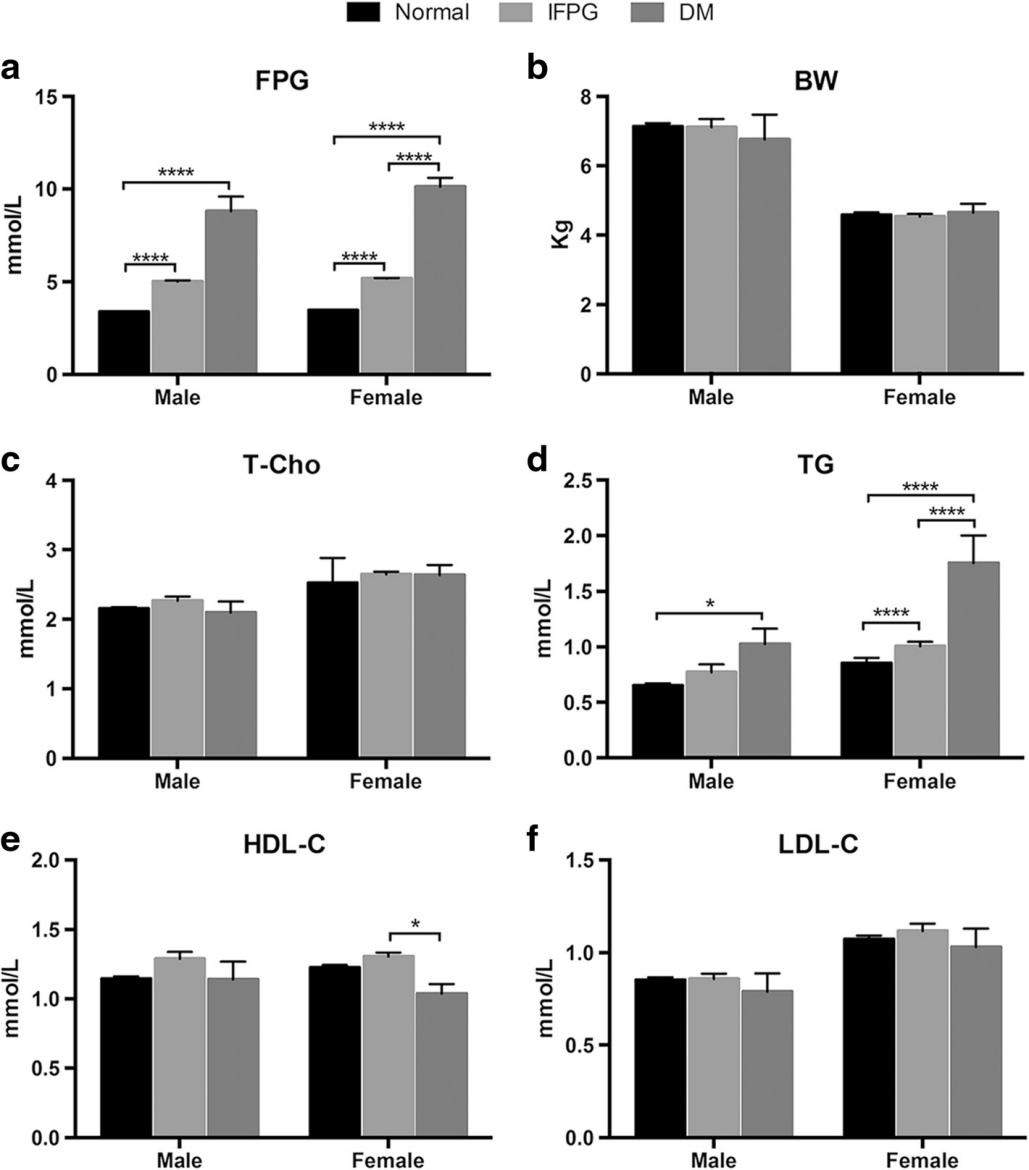
Sex-related differences between normal, IFPG and diabetic animals

When comparing between physiological conditions, the most notable differences were the levels of TG particularly in female animals, i.e. the highest levels of TG was observed in diabetic females followed by IFPG animals (Fig. 6d right three columns). In males, the significant differences were only seen between normal and diabetic animals (Fig. 6d left three columns). Another interesting discovery occurred in the diabetic female monkeys, it was found that they had the lowest level of HDL-C, in which the difference between female IFPG and diabetic monkeys was significant (*P* < 0.05) (Fig. 6e left two columns). No statistical significances were found in levels of T-Cho and LDL-C in male and female under the three conditions (Fig. 6c, f).

## Discussion

The result of the present study and others suggest that glucose metabolism slows with the aging process, which could lead to diabetes mellitus. More diabetic and IFPG monkeys were found in older and over-weight animals, although IFPG animals could be also seen in the younger cohorts. In humans, studies have found the impaired glucose tolerance occurs in approximately 25 % of people 65 years and older [14, 15]. The data from the current study also indicated that changes in FPG were not only age-dependent but also sex-dependent as well. More IFPG and diabetic female cynomolgus monkeys were found than their male counterparts. In this large cohort of monkey, it was more surprisingly seen as young as in 4 years old. Both sexes had elevated plasma glucose levels, which qualified as IFPG; this is equivalent to 12–16 years old human teenagers. The result apparently had agreements with some human studies, which have reported that the glucose tolerance decreases with advancing age. The primary cause of this age-related IFPG could be a result from tissue unresponsiveness to insulin [5, 16, 17].

The strength of the present study was using a large cohort of animals to examine age-related changes in the FPG; along with demonstrating that age- and sex-dependent factors can change FPG levels. However, the result was not supported by all nonhuman primate studies. For examples, Tigno and colleagues, on one hand, reported that FPG among the non-diabetic animals remained practically unchanged; indicating no age-associated changes in the FPG were seen in their study [18]. However, they found correlations between age and 2-h post oral glucose tolerance test (OGTT) and hemoglobin A1C (HbA1C) levels. The present study was focused on the fasting plasma glucose and not only found age-associated changes in FGP but also found the impaired FPG could occur even in as young as 4 years old monkeys, which was equivalent 12–16 years old human teenagers. Nevertheless, the overall result from the current study suggested that age is an independent factor for impaired fasting plasma glucose, which was inline with previous studies in human and nonhuman primates. Thus suggesting dysfunction of glucose metabolisms is one of the most common pathologies in aging humans [19] and NHPs [20].

One weakness of the present study was to find enough age-matched male cynomolgus monkeys to match the number of females used in the study due to the availability of older male monkeys. This could be the reason that more IFPG and diabetic female monkeys were identified. Other potential explanation may be due to sex differences in hormonal pathophysiology. A line of evidence suggests that T2DM affects women disproportionately to men [21]. Women with T2DM generally have poorer glycemic control and are less likely to reach the goals for hemoglobin A1c (HbA1c) compared with men. Women with diabetes have higher all-cause mortality [22–24].

Sex-related differences in levels of FPG were also found in nonhuman primates [7]. On the other hand, some community-based, cross-sectional studies revealed slightly different results. For example, a study conducted in Mauritius found that the prevalence of diabetes and the prevalence of coexisting IFPG and IGT (impaired glucose tolerance) were similar among men and women [25]. In addition, IFPG was found to be more common in men than in women; whereas IGT less common in men than in women [26].

The results from the present study further confirmed that some physiological measures change with age primarily including body weight, levels of TG, HDL-C and FPG. In fact, it has been known for decades that humans have plasma lipids changes that correlate with age that occur in both males and females [27]. Although the underlying reasons for the age-related changes are not completely delineated, several putative mechanisms have been proposed. For example, in rodents TG absorption can be impaired by as much as 50 % with advancing age; suggesting that aging is associated with changes in the absorptive capacity of the small intestine [28]. It could also suggest a decrease in TG absorption that could be underpinned by a decline in the secretion rate of the key digestive enzyme pancreatic lipase [29]. The current data agreed with some human studies in which the TG concentrations increase progressively in men, reaching peak values between 40 and 50 years, and decline slightly thereafter. In women, the TG concentrations increase throughout their life, and are higher in those using estrogens constantly [30]. Human and nonhuman primate studies reveal a trend in higher TG levels along with lower HDL-C levels correlate with aging. In fact, highest levels of fasting TG and lowest levels of HDL-C have long be regarded as potent cardiovascular risk factor in patients with and without diabetes mellitus [31–34].

Almost all lipoprotein levels, including HDL-C, are much lower at birth than at adolescence and increase during childhood [17]. HDL-C cholesterol in adults was shown to decrease with age in both men and women in prospective studies, most likely attributed to hormonal changes [35, 36]. Baggio and coworkers reported that the mean HDL levels of (both female and male) centenarians are 20 % lower than those of 65-year-old subjects [37]. On the other hand, older people and centenarians are unlikely to have very low levels of HDL; while mean HDL cholesterol levels did not vary or even increase with age in cross-sectional studies [38]. Decline in fasting HDL plasma concentration along with aging was found among all tested animals in the present study. Therefore, a negative and weak correlation between age and HDL-C was significant (*P* < 0.0001). Also in the present study, decline in levels of HDL-C appeared more sharply in male (*r*^2^ = 0.13, *P* < 0.0001) than female (*r*^2^ = 0.007, *P* = 0.0063) monkeys.

The relationship between lipid profile alarm and diabetes-associated complications has long been an area of interest due to the fact that diabetes can impair the utilization of lipids and lipoproteins which cause atherogenic dyslipidemia. This is one of the most important risk factors for the development of atherosclerosis in diabetic individuals [39, 40]. Dyslipidemia is a potent predictor of cardiovascular morbidity and mortality in diabetic patients. Based on American Diabetes Association (ADA) Standards [41], diabetic dyslipidemia is characterized by increased serum LDL, TG and decreased HDL. In this study, increased serum levels of TG were found in IFPG animals as compared with normal animals in both sexes; while a significantly decreased HDL-C was only seen in diabetic female monkeys. Overall results from the present study suggest that increases of serum lipoprotein along with aging were seen in both male and female animals in particular animals with elevated blood glucose. In a recent published study, Ali et al. reported that individuals with both diabetes and atherosclerosis had high levels of TC, TG, LDL, VLDL (very low-density lipoprotein) and low level of HDL in comparison to non-diabetic atherosclerotic and normal control individuals without any gender differences observed. Among all three age groups, lipoprotein abnormality was observed to be more frequent in females than in males [42].

This study for the first time highlighted age- and sex-effects on fasting plasma glucose and emphasized the relationship between elevated plasma glucose and lipid profiles in both male and females. The comparison of fasting plasma glucose and lipid profile in normal, IFPG and diabetic animals; and also age-associated differences between males and females, provide important information; such as IFPG can be found in cynomolgus monkeys in both male and female as young as 4 years old, even though they maintain normal body weight, and the relationship between FPG and TG. The nonhuman primate data being inline with some findings in humans indicates females are more prone to have elevated plasma glucose and fasting T-Cho, TG and LDL-C. However, female monkeys have higher levels of HDL-C compared with males as well. The overall result suggests cynomolgus monkey with naturally spontaneous elevated plasma glucose and lipoprotein levels, in particular, are useful models to study human metabolic syndromes and have the potentially to be used for screening new therapeutic strategies for treatment for T2DM and other metabolic diseases.

## Conclusions

The result, for the first time, demonstrated the similarities and differences in detail between male and female cynomolgus monkeys in relationship to age-related glucose and lipoprotein metabolisms, and differences under various physiological conditions. The detailed glucose and lipoprotein profiling should provide additional and important insights for prediabetic conditions. Cynomolgus monkeys appear to be an excellent model for translational research of diabetes and for novel therapeutic strategies testing to overt diabetes.

## Abbreviations

ANOVA, analysis of variance; FPG, fasting plasma glucose; HbA1C, hemoglobin A1C; HDL-C, high-density lipoprotein cholesterol; IFPG, impaired fasting plasma glucose; LDL-C, low-density lipoprotein cholesterol; OGTT. oral glucose tolerance test; T2DM, type 2 diabetes mellitus; TC, total cholesterol; TG, triglyceride
